# Opportunities to sustain matricentric models of community and person-centered care for perinatal substance use in the post *Dobbs* Deep South

**DOI:** 10.3389/fpsyt.2025.1646213

**Published:** 2025-10-08

**Authors:** Regan A. Moss, Emily Locke, Arianna Injeian, Kelli Stidham Hall

**Affiliations:** ^1^ Social, Behavioral, Population Health Sciences, Celia Scott Weatherhead School of Public Health and Tropical Medicine, New Orleans, LA, United States; ^2^ Anthropology, University of Alabama, Tuscaloosa, AL, United States; ^3^ Epidemiology, Celia Scott Weatherhead School of Public Health and Tropical Medicine, New Orleans, LA, United States

**Keywords:** person-centered, matricentric, post-*Dobbs*, community-based, perinatal substance use

## Abstract

Substance use during pregnancy is a growing public health concern, in part due to increasing rates of pregnancy criminalization that are heavily concentrated in the United States (US) Deep South. While existing public health models of care are designed to address substance use during pregnancy, these models often center the fetus rather than the pregnant/birthing person. We argue that patient and community centered models of care are needed to ensure pregnant people who use substances have access to respectful and safe care. We identify person-centered and community-care models for birthing people that rehumanize the birthing person and transform their subjectivity, moving from an object of medical intervention to a collaborator in their own care. By integrating matricentric feminist framings with the Dynamic Sustainability Framework (DSF) we build on theorizations of person-centered care to further invert the medical gaze, resist the governing of pregnant bodies, and proactively prevent self and other regulation of birthing persons. In doing so, we identify opportunities to sustain community-centered peer support specialist, substance use doula, and peer support group care models into the unique cultural contexts, healthcare settings, and policy climates of the post-*Dobbs* Deep South.

## Introduction

### Perinatal substance use in the United States

Substance use during pregnancy and the postpartum period is a growing public health concern. Accounting for 25% of all pregnancy-related deaths in the United States (US), substance use is a leading cause of maternal morbidity and mortality and is associated with a multitude of risks for adverse health and social outcomes for pregnant people and their offspring and families. Most pregnant people do not choose to begin using substances once they know they are pregnant, and those who have the capacity to choose to quit or abstain on their own usually do so (1). This is the key distinction between substance use and substance use disorder (SUD) (2021). Substance use is the consumption of substances that alter physical, emotional, or cognitive states, and SUD is a chronic disease characterized by a cluster of cognitive, behavioral, and physiological symptoms indicating that the individual continues to use substances despite significant substance-related problems (2). Similar to the general population, many genetic, environmental, psychological, biological, and socioeconomic factors contribute to a pregnant individual’s substance use and/or SUD (3). These include an individual’s history of trauma, stressful interpersonal relationships, and poverty (1, 4). The most frequent substances used during pregnancy are tobacco, alcohol, and marijuana, followed by opioids and cocaine (3, 5). Notably, the legality of these substances varies considerably across the US but narratives of criminality and morality during pregnancy may render all people who use these substances during pregnancy subject to surveillance and critique.

According to the National Survey on Drug Use and Health 2020 report, approximately 14.9% of the pregnant population in the US reported using tobacco in 2015. Of the substances listed under the “illicit” (i.e., illegal) category, cannabis was the most widely reported, accounting for 112,000 (93.3%) of the 120,000 pregnant people reporting any illicit substance use. Those who reported past-year cannabis use were more likely to report using other substances, including cocaine, opioids, and alcohol (2020). Substance use during pregnancy is associated with a range of adverse health outcomes for pregnant individuals and their neonates, including postpartum depression (6), preterm birth, maternal mortality, stillbirth, neonatal abstinence syndrome (NAS), and infant death (7). In a cross- sectional analysis of inpatient pregnancy hospitalizations from the Healthcare Cost and Utilization Project National Inpatient Sample from 2016 to 2020, Ragsdale et al. (8) found that SUD was an independent predictor of fetal growth restriction, antepartum hemorrhage, and preterm birth. From 2010 to 2017, maternal opioid-related diagnoses increased approximately 130% from 3.5 to 8.2 per 1,000 hospital deliveries, and NAS increased 83%, from 4.0 to 7.3 per 1,000 hospital deliveries (9). This increase coincides with increasingly restrictive barriers to care that are prominent in the South.

### Barriers to clinical care access and continuity

While evidence-based treatments for perinatal substance use exist, they are difficult to access for many systems-marginalized birthing people^1^ and women. Barriers to clinical care include fear/risk of stigma, discrimination, potential Child Protective Services (CPS) involvement, penalization or criminalization, custody challenges, limited socioeconomic resources and logistical supports, and lack of understanding of local policies and reporting mandates on part of healthcare providers and systems (10–12). State actors, including local police departments and CPS, rely heavily on substance use allegations as a basis for charging pregnant people with criminal child (fetal) neglect (13). This legal apparatus lends itself to excessive surveillance of pregnant persons from within the healthcare and justice systems (2024). Such surveillance can occur when individuals are drug tested without their knowledge or consent (2024). Universal substance use screening for pregnant individuals has been shown to improve perinatal health outcomes by facilitating early identification and treatment of SUDs (14). However, universal screening becomes a dangerous practice when it is weaponized by state-level actors to criminalize pregnant people (15). Non-consensual drug screenings and subsequent engagement with CPS can also be due to misinterpretations of federal and state Child Abuse Prevention and Treatment Act (CAPTA) requirements (16). This is why *consensual* drug screenings are critical, as non-consensual drug screenings and engagement with CPS extends throughout the perinatal period and the pregnant person’s behavioral health needs can become grounds for child abuse cases (National Advocates for Pregnant Women, 2024).

These forms of surveillance amplify social stigma surrounding substance use during pregnancy that is already rooted in a general societal judgement towards people who use substances and the harmful stereotype that individuals who use substances are “weak” and “undeserving” of care (1, 17). This stigma is compounded by narratives of *normative motherhood* ^2^ (i.e., cultural stereotypes of who/what the mythical ‘perfect’ parent is/does) (18), which characterizes the ideal pregnant person as selfless, devoted, self-sacrificing for the fetus/baby/child (18). At the expense of her own wellbeing, the normative mother dedicates her own life – even her own biological needs – to the infant. As such, addiction is framed “as weakness” (1, 17).

Narratives of the selfless mother can be internationalized and perpetuated by healthcare providers and others within the healthcare system, leading to increased referrals to child welfare services and removal of parental rights for those that are perceived as “unfit to parent” (1).

Stigma towards pregnant people who use substances is particularly salient in rural areas, where communities are tight-knit and confidentiality can be difficult to maintain (19). Birthing people and women report that in attempts to avoid detection from state actors, they will isolate themselves, skip treatment, or avoid treatment altogether (20). Further, they may avoid disclosing the need for care or hide their substance use while seeking prenatal care (21).

Roberts writes that the employment of the welfare system against Black patients (i.e., criminalization) is fundamental to the pathologization of families and birthing people/women who are not White and middle class (22, 23). Black birthing people/women are disproportionately impacted by non-consensual substance use screenings during pregnancy and receive harsher punishments and penalties for suspected substance use (24–26). Thus, the clinical setting can be a space of surveillance for pregnant people who use substances, leading to barriers in access to care, especially for Black birthing people/women and other communities of color intending to reproduce (12, 27).

In addition to legal mechanisms, stigma impacts help-seeking behaviors and healthcare behaviors through psychological mechanisms. For instance, stigma undermines self-efficacy, self-esteem and a pregnant individual’s belief that they deserve or are worthy and capable of accessing care and treatment. (1, 28). Thus, pregnancy may become a point of internalized guilt and shame, leading pregnant people to self-surveil their own behaviors, reconstructing the meaning they attached to their bodies, pregnancies, and psychological development as maternal beings (2021). This can lead to the avoidance of care (20). Self-surveillance and the embodiment of normative motherhood can manifest in worsened mental health outcomes, including reports of guilt and shame, which hinder recovery and health. Further, stigma isolates mothers from social benefits, support systems, provider networks, and community resources (28–30).

The internalization of *normative* motherhood can lead mothers to feel shame and embarrassment if they deviate from the norm. Mothers report feeling like a ‘failure’ when they do not match the idealized version of birthing people/mothers (31). When birth parents who use substances receive affirmation and recognition of their subjective experiences as real and understandable from others, they are more likely to seek support (31, 32). In doing so, mothers may look to individuals with similar lived experiences—specifically, other birthing people/mothers who used substances during their pregnancy and throughout the postpartum period. These shared experiences are validating in that they help birth parents/mothers understand they are not alone. In the absence of this support, mothers can feel shame, denial, and emotional isolation. These feelings translate to *in*action: mothers have difficulties seeking support, sharing needs, and may mask or obscure their needs from others (31). This may lead to elevated needs for mothers who use substances during pregnancy as this often requires clinical intervention. Mothers may become further isolated from care when they do not feel recognized by the healthcare system. In the instance that mothers are connected to care, they may desire feeling emotionally safe with providers (33).

Discrimination is also a barrier to care. Individuals who are Black, Latine, and/or uninsured experience higher rates of discrimination in healthcare settings when seeking treatment for substance use relative to their White and/or insured counterparts (34). Discrimination has tangible effects on experiences of care: patients who experience discrimination leave treatment prior to the necessary duration and report treatment as less helpful compared to those who are not discriminated against (34). Individuals on Medicaid are more likely to experience discrimination than those who are privately insured (35). This can compound with other barriers to care, including access to transportation and childcare, that prohibit individuals from seeking treatment (2015).

### Under prioritization and utilization of community and person-centered models of care

Increasing rates of pregnancy criminalization, which disproportionately impact Black birthing people and birthing people living on low incomes, challenge public health and cross- sectoral partners to reconsider the ways in which we respond to substance use during pregnancy. Birthing people are forced to navigate excessive surveillance in sociocultural and healthcare systems, which is often in tension with self-defined health needs and the subjective experiences of pregnancy. Use of opioids and other illicit drugs have not been well prioritized in broader efforts to prevent and reduce maternal morbidity and mortality, especially those that are women- and community-centered (36). The political climate highlights opportunities to invest and sustain matricentric, women- and community-centered models of care.

### Matricentric approaches to care

Narratives of normative motherhood demand that birthing people/mothers “efface [their] own subjectivity” to be a selfless protector and producer of the fetus (17). Narratives of normative mothering and the selfless mother have become embedded into clinical practices regarding substance use during pregnancy and as a result, fetal-centered models of care–which mirror trends towards pregnancy criminalization–have been prioritized by state legislators, public health departments, and the biomedical apparatus (36). Centering the birthing person and their subjective experience of motherhood, pregnancy, and substance use is necessary to adequately respond and prevent maternal death and suffering (2024). Evidence-based interventions exist to support person-centered models of care for improving access and quality of care for birthing persons who use substances. Among them, peer support specialists, support groups, and community-based doulas hold promise as supportive, ancillary models of care for pregnant people who use substances.

These models are particularly valuable now that the US Supreme Court has eliminated the constitutional right to an abortion (i.e., a ruling that took place on June 24, 2022 in a case titled *Dobbs v. Jackson Women’s Health Organization*) because peer support specialists, support groups, and community based doulas transverse both formal (e.g., clinical, institutionalized) and informal (e.g., community-based, locally situated) systems of care. This allows them to center the birthing person, bridge sectors of care, and expand access to treatment while prioritizing client confidentiality and safety. Furthermore, a matricentric feminist framing^3^ to care can help resist the harms of fetal-centered models which have led to, or at the very least excused, criminalization of substance use during pregnancy. Matricentric feminism has been applied in public health and related sectors (e.g., social work) to confront patriarchal ideologies which may structure services, policies, and practices (Epstein & Mulley, 2024).

The conditions of the Deep South are not static. Sustaining care requires attending to the ever-shifting public health infrastructure, increasing rates of pregnancy surveillance, and stark issues in access to care which continue to grow post-*Dobbs*. The Dynamic Sustainability Framework (DSF) is an Implementation Science (IS) framework which helps “[address] the paradox of sustainment amid ongoing change” (37, pp. 1). While interventions may remain evidence-based, their “fit” within the broader system of care and political climate may not be fixed. Thus, the DSF provides a foundation to refine and improve models of care so they remain reliable and accessible to patients/clients amidst an ever-changing landscape (37). As Chambers and colleagues describe (2013), change exists in the use of interventions over time and is impacted by the characteristics of practice settings and the broader system that establishes how care is delivered. The DSF model is composed of three layers: the intervention (layer 1) is nested within the practice setting (layer 2), which is nested within the ecological system (layer 3).

### Purpose

In this article, we explore the potential role of peer support specialists, support groups, and community-based doulas in mitigating the criminalization and surveillance of pregnant people who use substances. We situate their position as community-based caregivers in the post-*Dobbs* landscape, recognizing the dire need to rehumanize the birthing person as more than a subject of state-level control and public health intervention. We suggest that these models may serve as protective factors against the harms of fetal-centered care through their centering of each birthing person’s subjective experience. Further, we integrate our matricentric feminist framing with the DSF, providing an outlet for public health actors to theorize ways to implement, scale, and sustain evidence-based patient-and community-centered modes of care. The DSF framework offers insight into how actors across fields can problem-solve and adapt person-and community-centered care into the unique and precarious cultural contexts, healthcare settings, and policy climates of the post-*Dobbs* Deep South.

### Positionality

We bring together our voices across women’s and gender studies, public health, and medical anthropology to propose a matricentric framing to support birthing people who use substances during pregnancy. The first author brings experience in co-designing community- based and grassroots-led health interventions for/with systems-marginalized mothers that center their subjective experiences of motherhood. Her research has examined how well-meaning public health and clinical actors may further marginalize mothers by (unintentionally) perpetuating harmful narratives or stereotypes about their experiences. The deficits of public health interventions are especially stark in low-resourced and underinvestment areas, where birthing people have limited choice in the interventions they choose or have access to. Due to her upbringing in a low-resourced and conservative region of the Ozarks, she has worked to understand how public health interventions can help confront or further social stigmas for people living in rural communities. In addition, her lived experience in a low-resourced setting provides insight into opportunities to sustain models of care in spaces with a lack of infrastructure. She believes that matricentric feminist theory and maternal psychology can be applied in tandem with behavioral sciences to more aptly respond to the maternal health crisis.

The second author has been a community-based doula in [hidden for review] for four years. She has supported multiple clients with substance use disorder and has learned how to navigate complex clinical settings and legal systems. She is trained to provide trauma-informed doula care, mental health first aid, and postpartum support. As a PhD candidate in medical anthropology, she is committed to understanding the lived experiences of nurses, obstetricians, midwives, and doulas who provide care to pregnant people in hospital settings. She has conducted research in [hidden for review], where she helped identify strategies for increasing access to doula care and collaborative perinatal care across the state. She is a member of the Institute for Medicaid Innovation’s Doula Learning Action Collaborative for the state of [hidden], an intensive 3-year effort that will increase access to evidence-based community doula services for families who have Medicaid insurance coverage. Her current research explores the relationship between interprofessional collaboration, patient-centered care, and hospital-based doula care. She is committed to fostering collaborative, perinatal healthcare that respects the ancestral practice of midwives and doulas and centers the needs of birthing people.

The third author brings a medical anthropology and public health background alongside direct clinical experience supporting people navigating reproductive healthcare under criminalized conditions. Her commitment to this work emerged through providing emotional and clinical support to patients navigating and receiving surgical abortions, many of whom concurrently navigated substance use disorder. Currently, as a doctoral candidate in medical anthropology and recent MPH graduate, she continues community-based work as a medical assistant alongside supporting public health measures, in a local reproductive health clinic in the Deep South. This clinic transitioned from providing abortion services to focusing on comprehensive reproductive care post-*Dobbs*, serving primarily low-income, minority patients through an integrative care team. This direct experience of how criminalization reshapes healthcare delivery, combined with witnessing patients navigate substance use, pregnancy, and systemic barriers, drives her interest in research that centers pregnant and birthing people’s experiences and challenges punitive approaches to reproductive healthcare.

## Matricentric person- and community-centered models of care

### Ecological system: pre and post-*Dobbs* climate

Meeting the complex needs of birthing persons who use substances can present unique care challenges and broader sociocultural and political considerations within the perinatal care space. These challenges are magnified in the Deep South where punitive policies designed to police women and pregnant people restrict access to substance use treatment (38). For example, in 2006, Alabama passed *Chemical Endangerment of Exposing a Child to an Environment in Which Controlled Substance Are Produced or Distributed*, an act originally intended to protect children from the dangers of methamphetamine labs that has since been reinterpreted to prosecute pregnant people who test positive for controlled substances (39). In 2014, Tennessee explicitly authorized assault charges against people who used narcotic drugs during pregnancy (40). Although it expired in 2016, this “fetal assault law” has been re-introduced several times to Tennessee’s legislature in support of permanent implementation (2021). Similar laws have been introduced in South Carolina, Tennessee, Oklahoma, and Mississippi. As a result, nearly four in five criminal arrests of 1,379 pregnant people between January 2006 and June 2022 took place in Alabama (46.5%), South Carolina (13.05%), Tennessee (9.4%), Oklahoma (8.1%), and Mississippi (2.6%).

The *Dobbs vs. Jackson Women’s Health Organization* ruling has only served to heighten rates of pregnancy criminalization in the Deep South. In the year following the *Dobbs* decision (2023), pregnancy criminalization cases reached 210, a record high in a given year, and a major acceleration in the 1,600 cases from the 16 years preceding *Dobbs* (38). Punitive laws can turn routine patient-provider interactions into potential legal threats, in which pregnant people can be persecuted for seeking either prenatal care or substance use treatment (41, 42).

For pregnant people who use substances, healthcare encounters carry perpetual threats of criminalization, which can lead to potential risks of CPS involvement after childbirth (43). This transforms spaces of healing into sites of violence and criminalization, often against the clinical providers’ will. This state-sanctioned violence is a deterrent to treatment (44). Without treatment, pregnant people who use substances are at an increased risk of poor perinatal health outcomes, including fetal outcomes (43). This creates a public health paradox whereby heightened surveillance and criminalization undermines the outcomes it purports to protect.

Further, Medicaid restrictions exemplify how policy functions as systematic exclusion. Pregnancy verification, long waiting periods, and limited provider acceptance of Medicaid, create health barriers that disproportionately impact pregnant people who use substances (45). This will become an even deeper, ongoing challenge as the “One, Big Beautiful” Act plans to cut Medicaid by at least $600 billion–the largest cut Medicaid will have experienced–further restricting access at a time when more support is needed. Medicaid is the backbone of obstetrical care, covering 40.2% of births in the US (CDC, 2024), and foundational to the provision of behavioral health care within the US. The impacts of this bill will likely have stark consequences for pregnant people use substances, widening access to care and increasing the prevalence of maternity care deserts. Notably, regions experiencing behavioral health provider shortages in the US are often the same regions with limited access to obstetrical care. Even in spaces without provider shortages, pregnant people who use substances are more likely to be denied access to behavioral health services as compared to their non-pregnant peers (46).

### Hybrid practice setting

Community and person-centered models of care can serve as a potential buffer to this paradox. Such models often exist within and beyond the public health sectors, rendering them uniquely vulnerable to funding cuts but also uniquely positioned to support birthing people that experience marginalization. In many cases, they function as hybrid practice settings, in which they bridge gaps between clinical and community support. The hybrid practice setting provides a unique opportunity for person-centered and community-centered models of care as these models are increasing in availability for people within and outside of formal care settings; however, they may also be underinvested as they are not one sector’s sole responsibility.

This is particularly important, given that many pregnant people who use substances must also navigate a dearth of perinatal healthcare resources. Since the 1980s, maternity care deserts (i.e., counties where there is no access to birthing hospitals, birth centers offering obstetric care, or obstetric providers) have increased in prevalence (47).

## Person and community-centered models of care

The embodied experiences addressed within community-centered models of care encompass the full spectrum of maternal experiences that can be criminalized in the Deep South. These experiences are shaped by racialized and classed norms of “good mothering” that create additional trauma for birthing people who use substances, particularly Black and minoritized women who face intensified surveillance and criminalization (1, 48). Gender-specific and trauma-informed care recognizes these intersections and provides opportunities for reconstructing maternal identity outside of dominant narratives that reduce birthing people to vessels for fetal protection rather than whole human beings deserving of care and support (49–51).

Importantly, therapeutic monitoring within community-centered care creates a fundamentally different dynamic than punitive state surveillance systems. Professional guidelines within community-centered care models are designed to support maternal goals and protect maternal interests rather than police maternal behavior or enforce compliance with external standards. This approach explicitly addresses how criminalization systems target birthing people and provides collective strategies for resistance and protection (20).

Community-centered care providers, including peer support specialists and community-based doulas, can deliver care through both group based and individual modalities that embody harm reduction principles—respecting people seeking care and providing opportunities to set realistic goals in safe environments that prioritize relationships over surveillance (52). Community-centered care models indicate that enforcing harm reduction while focusing on trauma-informed and gender specific care is highly beneficial and leads to addressing social determinants of health through non-punitive, continuous care approaches rather than taking an abstinence-only approach (50, 53, 54). Matricentric community-centered care approaches center the pregnant person’s subjective experience and resistance to oppressive systems (55).

Community-centered models of support have been found to be impactful in integrative, trauma informed care for pregnant people who use substances (3, 56). This is not to say that we propose community-centered models replace clinical care; rather, they can serve as a collaborative, supplemental measure for improving outcomes (57).

In integrative care teams or community-centered care teams, many pregnant people who use substances find increased engagement and accountability. Notably, there is community created through these modalities, an integral piece of care that can encourage continuation in substance use treatment as well as support in and out of group sessions (58). Community-centered care structures are beneficial for both people who use substances while pregnant and postpartum. Trauma-informed spaces where pregnant and postpartum people have the space and security to disclose their experiences increases self-efficacy and can lead to more accountability to pursue and follow through with substance use disorder treatment (59, 60). Community-centered models provide crucial advocacy support and practical guidance for navigating hostile systems while maintaining focus on maternal wellbeing and family preservation. In a post-*Dobbs* landscape, pregnant people who use substances are most targeted. When options for abortion and contraception are cut drastically, especially in the Deep South, pregnant people who use substances, specifically people of color, will be most targeted for criminalization and other punitive measures (61, 62).

*Peer Support Specialists:* “It’s the sharing of experiences with other women which really matters (63).”

Peer Support Workers or Peer Support Specialists (PSS) in the context of substance use related care are certified professionals who often have lived experience with substance use and parenting/pregnancy (64). In addition to lived experience, PSS may become certified by working with birthing people who use substances. While PSS are certified, they are not necessarily formally employed by healthcare systems, positioning their role as abridged between formal and informal public health spaces. For instance, PSS may be tied to a nonprofit organization or employed by a public health agency. Likewise, mothers report that PSS can be a form of formal support or serve as an adjunct to formal support (65).

PSS have positive benefits especially for mothers who experience social stigma within and outside of the clinic. Mothers who use substances may become isolated from other mothers. They report experiences of isolation and disengagement from care when their mental health needs are not “shared by other mothers”, leading to limited understanding among “peers” or other mothers without the same needs (31). PSS help build social cohesion and affirm positive psychological developments of maternal identity. Clients/patients attribute health improvements to “receiving empathetic listening, acceptance, affirmation and normalization” (66). The relationships may mitigate the harms of social stigma (67).

PSS care and support may vary as it is centered on the birthing person’s unique needs. Support can be grouped into four key domains: emotional, informational, instrumental, and affiliations (64). PSS support facilitates the development of formal and information supports who can instill confidence, assist in goal setting, and serve as advocates, mentors, and facilitators for the resolution of issues related to health and well-being. PSS are necessary for enhancing and improving the health of individuals with emotional, behavioral, and/or co-occurring disorders (68).

The PSS model recenters the mother in care and recognizes her subjective experience with substance use during pregnancy. PSS is believed to be effective as it fosters shared affiliation, a deep understanding of [shared] experiences, and a sense of belonging—all key to recovery from behavioral and mental health needs (68) PSS recognize the pregnant person’s subjective experience of using substances, which serves as the foundation for helping them navigate the clinical care space.

PSS address systems of marginalization while ensuring the client/peer is still connected to needed care, increasing adaptive help-seeking behaviors. In addition, birthing people with PSS support report increases in self-efficacy, trust, and safety, (31) Other benefits include: reduced substance use and SUD relapse rates, improved relationships with treatment providers and social supports, increased treatment retention, and greater treatment satisfaction (69).

PSS may be integrated into healthcare teams. Olding et al. (31) describes how integration of PSS into care teams shifts relations among clinical staff and may transform clinical practices towards more patient centered models of care. Providers can have deeper engagement with reflection and mindfulness to confront social stigma and shift towards patient-centered models of care and away from criminalization of health behaviors (3). Mothers who use substance report these changes to be beneficial (31). Integration has unique potential in the post-*Dobbs*, Deep South where care is fragmented. However, if integrated, peer support specialists must be integrated equitably and supported adequately (31), which may be unique to sustain in care deserts and areas where public health has experienced mass disinvestments. When PSS are not integrated into healthcare systems, they may express challenges in “establishing credibility [ … ] managing system barriers”, perceived stigmas from clinical care providers, and navigating clinical boundaries (69, 70). Clinical boundaries may be challenging as PSS report unclear job descriptions (69, 71). Other barriers include low compensation and a lack of investment in the PSS workforce, which leads to high burnout and turnover rates (72, 73). While PSS experience high rates of burnout and low compensation, they report a sense of social support and satisfaction in their job, highlighting opportunities to sustain their care with meaningful public health investment (74).

Group based PSS can additionally be delivered both in person and virtually, with particular advantages for birthing people in rural Deep South areas where services are limited, and criminalization fears are heightened. Virtual delivery provides increased accessibility while reducing exposure to surveillance systems that may be embedded in formal healthcare settings (75). In-person PSS groups offer stronger community building while providing direct support for navigating local systems that may be hostile to birthing people who use substances.

### Community-based doulas

Doulas are globally recognized perinatal healthcare professionals found across many different cultures and traditions. The role of a doula has, in many respects, existed throughout human history: individuals have supported one another throughout the reproductive life course for millennia (76). In the US, doulas are most commonly recognized as trained, non-clinical perinatal healthcare professionals who provide emotional, physical, and informational support to birthing people during the prenatal, intrapartum, and postpartum period (77). While birth doulas are the most utilized by pregnant persons, there are several other types, including fertility, postpartum, bereavement, and full-spectrum doulas (78). Full-spectrum doulas offer support throughout the entire spectrum of pregnancy, from preconception to birth, abortion, miscarriage, adoption, and the postpartum period (79). Support can be informational, emotional, and/or physical, depending on the individualized needs of each client (2022). Many birth and full-spectrum doulas are community-based, meaning they support low- income families for little to no cost (80). Most community-based doulas are employed by or volunteer with local non-profits (81). In some states, community-based doulas are reimbursed through Medicaid and provide services to Medicaid- eligible families through community doula hubs and/or hospitals that offer billing support for the doulas.

Community-based doulas have an expanded scope of care relative to private-practice doulas because they spend considerable time helping low-income clients access safe housing, food security, and comprehensive systems of social support (80 82). Many community-based doulas are racially and linguistically concordant with their clients (2025). Such concordance can help Black, Brown, Indigenous, Latine, Medicaid-eligible, and/or substance-involved individuals feel safe and understood when encountering a healthcare system that has historically limited their access to respectful maternity care (83, 84). This sense of safety can also be facilitated by doulas who are not from the same community as their clients but engage in cultural humility and structural competence, i.e., the process in which providers analyze, challenge, and intervene on cultural issues through a social- ecological lens (83, 85).

An emergent body of literature suggests that structurally competent, community-based doulas can and should be leveraged as a first line of defense in identifying substance use challenges among pregnant persons (86). Community-based doulas center the subjective experience of the pregnant person, and in doing so, build trusting relationships with their clients (84). Trusting relationships may lead pregnant people who are substance-involved to disclose their substance-use to their doula before seeking treatment, allowing the doula to facilitate access to care (86). Substance use screening and treatment are beyond the scope of a community-based doula; however, an opportunity exists to leverage their position as relationship-oriented, perinatal healthcare professionals (2024).

Community-based doulas can refer clients to inpatient or residential treatment for pregnant people, clinics that offer suboxone (buprenorphine/naloxone) and Subutex (buprenorphine) treatment, and local support groups (87). This is critically important in the Deep South, where some clinics may surveille and criminalize pregnant people more than others, usually in fear of being criminalized themselves (88). Community-based doulas can guide their clients towards treatment centers with reputations for protecting pregnant people from criminalization (2023). They can also attend prenatal and postpartum appointments with their clients to facilitate interprofessional communication and provide a sense of emotional safety (89, 90). This type of support has the potential to enhance experiences of dignity and respect for pregnant people who use substances in healthcare settings while also minimizing sites of surveillance and criminalization in socio-political regions like the Deep South. However, community-based doulas must navigate a patchwork of licensing, certification, and regulatory policies that are not always evidence-based and can exclude culturally specific practices (91).

### Sustaining care models

The DSF framework gives insight into how actors across fields can problem-solve and adapt person- and community-centered in the unique cultural contexts, healthcare settings, policy climates of the post-Dobbs Deep South. In review of these models, there are clear opportunities for these models of care to be sustained, requiring action by legislators, clinical staff, public health actors alike.

### Sustaining interventions: ecological system

PSS and doulas are not strictly within one sector. As a result, their support may fall to the wayside. While the value of their care lies in part to their fluidity across sectors, formalizing the support they receive across sectors is critical. Adequate compensation for their labor is a critical step in sustaining the workforce. As 40.2% of births in the US are covered by Medicaid (92), many non-clinical, perinatal healthcare professionals (e.g., doulas and PSS) have articulated a desire for Medicaid coverage (93). However, there is not one best-practice model for Medicaid reimbursement of these services (93). Doulas and PSS across the US have reported the need for increased reimbursement rates and pathways to refine and improve Medicaid coverage models (94). The Institute for Medicaid Innovation (IMI) Doula Learning and Action Collaborative is currently working with seven states to identify best practices in an effort to address low or non-existent reimbursement rates and poor compensation, which can lead to burnout, distress, and mental health challenges (95).

Ensuring these models of care are covered by Medicaid is critical to support perinatal healthcare professionals and clients/patients. Many states have taken to reimburse these care providers and illustrate high returns on their investment, including improved perinatal health outcomes (96). Medicaid coverage for doula services is complex due to state-by-state variability in coverage, requirements, and reimbursement rates. While some states have implemented Medicaid reimbursement for doulas, challenges such as administrative barriers, inconsistent policy implementation, and low compensation limit widespread access and participation. Additional investment in these workforces includes continued education pathways, specifically surrounding best practices to supporting clients with substance use related needs.

### Sustaining interventions: practice and intervention settings

One of the key barriers to sustaining care is clinician-facilitated discrimination or judgement towards non-clinical support professionals, such as doulas and peer support specialists, that are part of the care team. As Scanell (70) and Eddie et al. (69) illustrate, paraprofessionals navigate stigmas and challenges establishing credibility within care settings. A multi-disciplinary commitment to collaborative care models can help address stigmas and challenges in integrated PSS and doulas into the clinical care environment.

Public health and healthcare administration should ensure clinical staff are trained in the benefits of multidisciplinary care teams and confident in applying interprofessional communication stigmas. Not only should clinical staff know the benefits of these other providers, but they should collaborate with them and strengthen the continuity of care as a result. Enhanced partnership working is foundational to care continuity across the perinatal mental health care pathway (33). Members of the care team can work with PSS and doulas to co- create clear job descriptions to collaborate with other members of the care team while providing the flexibility to remain person-centered and adaptive to the needs of the client.

### Resisting criminalization

While many actors have worked to integrate these care models at least partially into clinical settings, the post-*Dobbs* climate challenges the field to consider how formalization of a role within a clinical care setting may lead to criminalization. Healthcare systems should work to integrate PSS, doulas, and support groups into referral pathways and ensure the perspectives of PSS and doulas are honored by staff. The Deep South is not the only region of the US experiencing stark barriers in access to care amidst growing maternity care deserts and behavioral health shortages (97). These models of care will have benefits for others in different cultural contexts and may even provide the foundation to proactively prevent heightened pregnancy criminalization in other regions of the US.

## Conclusion

Many pregnant people in the post-*Dobbs* Deep South face unmet care needs for a broad range of obstetric and behavioral conditions. Birthing people who use substances are forced to navigate surveillance, criminalization, and stigma, which leads to inaccessible care, poor mental health, and further marginalization within care systems. Excessive surveillance within clinical settings undermine public health efforts to minimize pregnancy criminalization. We argue that future efforts can include matricentric, community-centered models of care that do not force birthing people to sacrifice their maternal role and needs. When the care model is fetal-centered, the birthing persons who is substance-involved is more likely to experience discrimination, stigma, and avoidance of care. Recognizing the call to “embrace the culture of a learning healthcare system” (66), we integrate matricentric feminist framings with the Dynamic Sustainability Framework to underscore how matricentric- models of care are necessary for sustaining matricentric, community-centered care models in the post-*Dobbs* Deep South.

Birthing people who use substances desire and deserve recognition, affirmation, and social support in order to successfully engage in treatment and have adaptive transitions into their experience as a mother. Pregnancy, childbirth, and the postpartum are not merely sites for biomedical intervention, but rather a critical point in matrescence. Supporting birthing people in their transitions to childbirth and/or parenthood requires recognizing them as whole people, rather than a biomedical subject. Peer support specialists, support groups, and substance use doulas all inherently center the birthing person as they are relational care models and define human connection as the lever for changes in health status.
